# Effect of offline bullying on online unethical behavior among college students: the mediating roles of relative deprivation and anger rumination

**DOI:** 10.3389/fpsyg.2024.1407157

**Published:** 2024-08-06

**Authors:** Ying Lin, Zhiwen Tang, Qiang Xing

**Affiliations:** ^1^School of Education, Guangzhou University, Guangzhou, China; ^2^Institute of Entrepreneurship, Guangdong Women's Polytechnic, Guangzhou, China; ^3^School of Management, University of Electronic Science and Technology of China, Zhongshan Institute, Zhongshan, China

**Keywords:** offline bullying, online unethical behavior, anger rumination, relative deprivation, college student

## Abstract

This research delves into the correlation between offline bullying and online unethical behavior among college students; and examines the potential mediating influences of anger rumination and perceived relative deprivation. The findings suggest that anger rumination, perceived relative deprivation, offline bullying, and online unethical behavior exhibit significant positive correlations with each other. Offline bullying is a strong predictor of online unethical behavior among college students, with the dual mediating effects of perceived relative deprivation and anger rumination on the relationship between offline bullying and online unethical behavior. This suggests that offline bullying directly influences college students' online unethical behavior and also influences it indirectly through anger rumination and perceived relative deprivation.

## 1 Introduction

Online unethical behavior is generally considered as any behavior that violates moral norms or principles in the context of online interactions (Ullrich et al., 2020), including cyberbullying, violation of privacy, spreading misinformation, etc.

The subject of online unethical behavior has its roots in the real experiences of individuals, and their actions in the virtual space of the internet are still influenced by their offline experiences. General Strain Theory proposes that when individuals encounter aggression or negative experiences, these incidents can lead to antisocial behaviors or misbehavior (Agnew, 1992). The General Model of Aggression suggests that individuals who are bullied in real life are also vulnerable to online bullying and tend to show more aggression and hostility after experiencing online bullying (Kowalski et al., 2014). Individuals who hid in the online space often feel more braver and powerful to resist and retaliate against bullying, which may lead them to commit unethical act online to maintain their psychological balance and regain their self-confidence (Fan et al., 2023). Those who reported experiencing severe bullying were significantly more likely to engage in academic cheating, plagiarism, and visiting inappropriate websites compared to their peers who were not bullied (Doe et al., 2019).

Offline bullying can serve as a risk factor for later online unethical behavior. Individuals who are bullied offline often perceive themselves as being in a lower status (Mummendey et al., 1999), leading to feelings of injustice and dissatisfaction. These negative emotions can encourage violent behavior (Kunst and Obaidi, 2020), which arises from a feeling of relative deprivation. Relative deprivation results in a cognitive distortion in the way individuals perceive their circumstances, and this distortion serves as a catalyst for the emergence of angry thoughts (Mummendey et al., 1999).

After being bullied offline, individuals often dwell on past negative events and stressful experiences, which will leading to persistent feelings of anger and anxiety (Borders, 2020), their anger rumination will be reinforced (Hamer and Konijn, 2016). It also awakens the individual's sense of aggression and puts them in a state of anger and resentment causing the individual to develop a state of aggressive and inappropriate behavior repeatedly (Pedersen et al., 2011), leading to increased aggressive behavior (Watkins and Roberts, 2020; Camacho, 2021).

Anger rumination is understood as the tendency to continuously think about and relive anger-inducing events (Sukhodolsky et al., 2001), which leads to more enduring and extensive harm to individuals, consequently resulting in an increase in unethical behaviors. Onlinespace presents the features of reproducibility, persistence, and all-weather accessibility (Kiesler et al., 1984; Kowalski et al., 2014), intensify anger rumination (Jose and Vierling, 2018). Research by Love H. et al. (2023) further emphasizes that individuals who experience frequent bullying are likely to exhibit higher levels of anger rumination.

Whether in cyberspace or offline real space, the decision-making process of ethical behavior is always based on the individual in real society as the subject of ethical behavior. The topic of online unethical behavior lives in real space, but also on the Internet for interpersonal communication, learning and life in the network. The environment in which the problem of online unethical behavior is located must consider both the real space and the cyberspace in which the individual lives, and the moral events that take place between the two will influence each other.

How stressful events in the offline space affect an individual's online ethical behavior in the online space is worthy of research and requires further study. How does offline bullying behavior as a typical negative event affect individuals' online ethical behavior? By examining the connection between offline bullying and online unethical behavior, we can gain a deeper understanding of the underlying causes that lead to online unethical behavior. At the same time, it emphasizes the continuity between the real-world and the digital space, provides a basis for a comprehensive understanding of individuals' offline and online misbehavior. Developing more targeted and effective interventions and prevention measures for policy makers, educators, and mental health professionals is critical. Enriches applied research on General Strain Theory (GST) and the General Model of Aggression (GMA) in the field of ethics.

**Figure 1:**
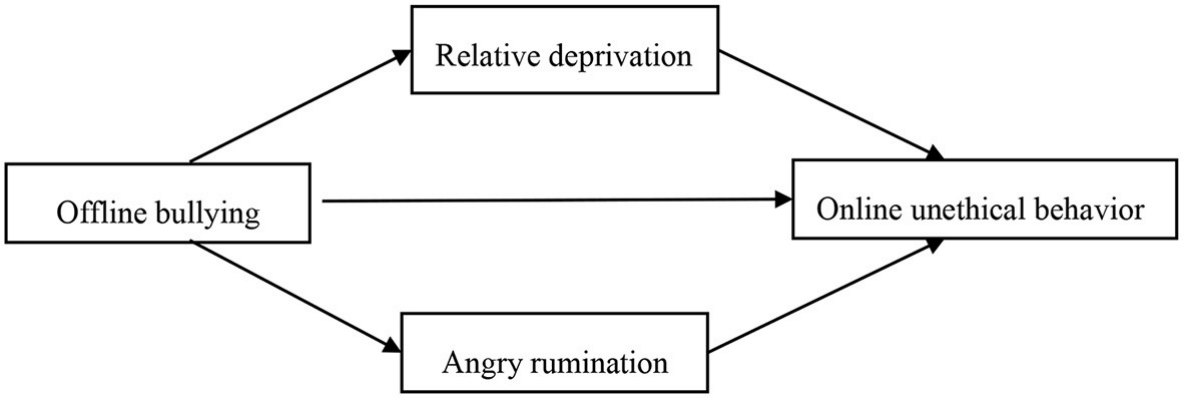
Dual mediation model hypotheses between realistic bullying and online unethical behavior.

How does offline bullying influence individuals' online unethical behavior? What kind of relationship exists? The hypothetical model proposed in this study is shown in Figure 1.

## 2 Method

### 2.1 Subject

In this study, stratified sampling was used to select five types of universities in Guangdong Province, such as 985-project universities, 211-project universities, double-first-class construction universities, other public universities, and private universities, and one school was randomly selected from each of them, with a total of five schools for the questionnaire survey. A total of 412 questionnaires were distributed, and invalid questionnaires in the recovered questionnaires were removed based on (1) respondents presenting obvious regularity in answering, such as the cycle of “12,345”; (2) respondents choosing extreme options for all questions, such as choosing the highest score or the lowest score for all of them; and (3) respondents completing the questionnaires in too short a period of time, and 2 min was used as a pre-curated and reasonable answering time in the present study, and data with < 2 min of answering time were removed. Finally, 402 valid questionnaires were obtained. The analysis included 173 male participants, *M* = 20.48, SD = 1.78, the age range was [19, 22]. Approval for the study was obtained from the university's academic board, and participants voluntarily signed a written informed consent before completing the questionnaire.

### 2.2 Measure

Participants completed an author-compiled demographic measure, assessing gender (identifying as male, female), age (in years), and grade (identifying as 1st, 2nd, 3rd, 4th).

#### 2.2.1 The bullying behavior scale

The scale consists of 14 items, which include two subscales, verbal bullying and relational buying (Liu, 2006). Participants rated each item on a 5-point Likert scale (1 = never to 5 = always), and higher mean scores indicate more bullying behaviors experienced. The scale has good reliability and the Cronbach's alpha coefficient for the scale in this study was 0.902.

#### 2.2.2 Anger rumination scale

The scale is a 19-items scale that includes four dimensions: anger after the fact, memory of anger, revenge thoughts and understanding the cause (Luo et al., 2011), the Cronbach's alpha coefficient was 0.87. Participants rated each item on a 4-point Likert scale (1 = occasionally or no to 4 = always). In this study, the Cronbach's alpha coefficient was 0.911.

#### 2.2.3 Relative deprivation scale

The 4-items scale (Ma, 2012) is one of the commonly used scales by academic researchers, which is widely recognized by scholars. Participants rated each item on a 6-point Likert scale (1 = strongly disagree to 6 = strongly agree). In this study, the Cronbach's alpha coefficient was 0.825.

#### 2.2.4 Online unethical behavior scale

The scale is a 16-items scale that includes four dimensions, network attack behavior, network infringement, false network information and network pornography, Validated to have good reliability (Lin and Tang, 2024). Participants rated each item on a 5-point Likert scale (1 = Never to 5 = Always). In this study, the Cronbach's alpha coefficient was 0.890.

## 3 Data analysis and results

### 3.1 Common method deviation

This study employed Harman's single factor test to examine common method deviation. All items from the four scales were subjected to exploratory factor analysis. The analysis revealed eleven factors with eigenvalues >1, exceeding a single factor. Additionally, the variance explained by the largest factor was 28.71%, which was below the 40% threshold. Therefore, the results indicate that common method deviation is not present in this research.

### 3.2 Correlation analysis between the various variables

The results of the correlation analysis between the variables are shown in Table 1, showing significant positive associations between offline bullying, relative deprivation, angry rumination, and online unethical behavior, *p* < 0.01.

**Table 1:** Correlation analysis between the various variables.

	**Offline bullying**	**Relative deprivation**	**Angry rumination**	**Online unethical behavior**
Offline bullying	1			
Relative deprivation	0.518^**^	1		
Angry rumination	0.448^**^	0.577^**^	1	
Online unethical behavior	0.557^**^	0.636^**^	0.546^**^	1

^**^p < 0.01.

Offline bullying was found to be significantly and positively correlated with relative deprivation, anger rumination, and online unethical behaviors (*r* = 0.5218, 0.448, and 0.557; *p* < 0.01). This indicates that the more severe the offline bullying experienced, the higher their levels of relative deprivation, anger rumination, and engagement in online unethical behaviors. Furthermore, relative deprivation was significantly and positively correlated with both anger rumination and online unethical behaviors (*r* = 0.577 and 0.636; *p* < 0.01), suggesting that higher levels of relative deprivation among college students are associated with increased anger rumination and online unethical behaviors. Additionally, angry rumination was significantly and positively correlated with online unethical behaviors (*r* = 0.546; *p* < 0.01), indicating that higher levels of anger rumination are associated with increased online unethical behaviors.

The findings suggest that offline bullying may lead to increased relative deprivation among college students, which in turn may trigger more anger rumination and ultimately result in higher levels of online unethical behaviors. Addressing the issue of offline bullying among college students could help in mitigating the emergence of negative emotions and behavioral problems.

### 3.3 Analysis of the dual mediation effects of relative deprivation and anger rumination

This study utilized the PROCESS 4.0 plug-in for SPSS software to conduct its analysis, with offline bullying serving as the independent variable, and online unethical behaviors as the dependent variable. Relative deprivation and anger rumination were examined as mediating variables. Model 4 was selected to investigate whether relative deprivation and anger rumination mediate the effect of offline bullying on online unethical behaviors. The bootstrap sampling method was employed for testing, and specific results are presented in Table 2.

**Table 2:** Model test of the mediation effect.

**Predictive variable**	**Explaining variable**	***R* ^2^**	***F***	***B***	***t***
Offline bullying	Online unethical behavior	0.556	179.54	0.524	12.01^***^
Offline bullying	Relative deprivation	0.518	146.77	0.719	8.11^***^
Offline bullying	Angry rumination	0.448	100.36	0.317	16.44^***^
Offline bullying	Online unethical behavior	0.714	138.27	0.247	2.34^***^
Relative deprivation				0.245	3.48^***^
Angry rumination				0.311	17.37^***^

^***^p < 0.001.

Offline bullying had a significant direct effect on online unethical behavior (R^2^ = 0.556, *B* = 0.524, *p* < 0.001). This suggests that even after controlling for the effects of relative deprivation and angry rumination, offline bullying still has a significant positive impact on online unethical behavior. Both relative deprivation (*R*^2^ = 0.518, *B* = 0.719, *p* < 0.001) and angry rumination (*R*^2^ = 0.448, *B* = 0.317, *p* < 0.001) are significantly predicted by offline bullying, suggesting that offline bullying contributes to these mediating variables. When considering the combined effect of offline bullying, relative deprivation, and angry rumination on online unethical behavior, the explanatory power of the model increases significantly (*R*^2^ = 0.714), with all predictors showing significant effects (*B* = 0.247 for offline bullying, *B* = 0.245 for relative deprivation, and *B* = 0.311 for angry rumination).

Further test for the mediation effect between the variables, as shown in Table 3, both relative deprivation and angry rumination serve as significant mediators in the relationship between offline bullying and online unethical behaviors. However, the direct effect of offline bullying remains substantial, accounting for nearly half of the total effect. Relative deprivation partially mediates the relationship between offline bullying and online moral anomie behaviors, with a mediation effect value of 0.176. This indicates that 33.65% of the impact of offline bullying on online moral anomie behaviors is due to the mediation effect of relative deprivation. Similarly, angry rumination also plays a partial mediating role in the relationship between offline bullying and online moral anomie behaviors, with a mediation effect value of 0.100, meaning that 19.12% of the impact is mediated by angry rumination. Thus, both relative deprivation and angry rumination partially mediate the relationship between offline bullying and online moral anomie behaviors. Offline bullying influences online moral anomie behaviors through the mediation effects of both relative deprivation and angry rumination.

**Table 3:** Mediation effect of relative deprivation and angry rumination.

	**Efficiency value**	**Boot BI lower limit**	**Boot BI upper limit**	**Relative value**
Total effect	0.523	0.446	0.599	
Direct effect between offline bullying and online unethical behaviors	0.247	0.170	0.324	47.23%
Mediation effect of relative deprivation	0.176	0.131	0.248	33.65%
Mediation effect of angry rumination	0.100	0.056	0.160	19.12%

## 4 Discussion

### 4.1 Offline bullying is positively correlated with online unethical behavior

The results of this study demonstrate that offline bullying has a positive correlation with online unethical behavior. This finding is consistent with the research which concluded that students who are often bullied in real life are more likely to exhibit inappropriate behavior on the internet (Martins, 2021; Mazzone et al., 2021), indicating a vicious cycle of aggression.

The anonymity of the internet and the lack of moral cues make it easier for individuals who are bullied in the real world to express their anger and other negative emotions online. Victims of bullying often experience feelings of powerlessness, anger, and frustration, which they may channel into the digital realm where they feel less inhibited. The anonymity afforded by the internet plays a crucial role in this transformation. The lack of immediate, real-world consequences allows individuals to express negative emotions more freely and engage in behaviors that they might avoid in face-to-face interactions.

The anonymity of the internet diminishes the perception of accountability, leading to moral disengagement. This cognitive process allows individuals to rationalize unethical behavior by diffusing responsibility. Online platforms often lack clear moral cues, which makes it easier for individuals, especially those with prior experiences of bullying, to justify their unethical acts as retaliation or self-defense.

The internet's structure also facilitates the spread of unethical behaviors. The decentralized distribution of moral information and the bidirectional interaction inherent in digital communication can quickly transform personal grievances or ethical lapses into collective moral participation. This collective dynamic often results in the diffusion of responsibility, where individuals feel less personally accountable for their actions. The presence of a group engaging in similar behaviors reinforces the notion that such actions are acceptable.

Moreover, the phenomenon of moral disengagement is amplified online. Individuals often position themselves on a moral high ground, which leads them to criticize or attack others, modeling the behavior they have observed or experienced in bullying scenarios. The mentality of “if others can do it, so can I” becomes prevalent, further normalizing unethical behavior in the digital sphere.

### 4.2 The mediating role of relative deprivation

The findings of this study elucidate the mediating effect of relative deprivation on the relationship between offline bullying and online unethical behavior among college students. Relative deprivation intensifies individuals' negative emotions, which then propel them to engage in online deviant behaviors (Wang et al., 2023). Individuals frequently bullied in real life tend to experience higher levels of relative deprivation, making them more prone to exhibiting unethical behavior online (Li et al., 2023).

In the unique context of the internet, such behaviors can become even more uncontrollable. Experiences of offline bullying are intertwined with an escalation in online unethical behavior. Students who endure frequent bullying in real life are more susceptible to higher levels of relative deprivation (Liang et al., 2023). This is because continuous exposure to bullying episodes can lead to a persistent state of perceived injustice and social inferiority. When students find themselves entrapped in these adverse scenarios without feasible escape routes, feelings of despair and failure take root. Their emotional equilibrium is disturbed, leading to heightened feelings of anger, frustration, and helplessness. These emotions can override an individual's self-regulatory mechanisms, causing them to lose control and engage in online unethical behaviors.

In conclusion, the mediating role of relative deprivation provides a new explanatory perspective on the observed link between offline bullying and online unethical behavior among college students. By intensifying negative emotions, relative deprivation acts as a catalyst that propels students from adverse real-life experiences to deviant online behaviors. Understanding this mediating role highlights the need for targeted interventions that address both the immediate consequences of bullying and the underlying feelings of injustice and disadvantage. Such interventions could potentially mitigate the emotional disturbances caused by bullying, thereby reducing the likelihood of subsequent unethical behavior online.

### 4.3 The mediating role of anger rumination

The findings of this research indicate that anger rumination significantly mediates the relationship between offline bullying and online unethical behavior among college students. Individuals frequently bullied in real life tend to exhibit higher levels of anger rumination (Love S. et al., 2023), When an individual's accumulated anger reaches a boiling point without an outlet, they are highly likely to engage in extreme behaviors online. This phenomenon can be understood through the lens of emotional overflow, the point at which accumulated emotions seek release. This is because the psychological repression, grievances, and anger of those frequently bullied in real life often remain unaddressed in their daily lives, making the internet a more appealing outlet for them. Consequently, they are more prone to engage in unethical behavior online compared to their peers.

The mediating role of anger rumination in the relationship between offline bullying and online unethical behavior underscores the complexity of these interactions. Unresolved anger, psychological repression, and the allure of the online world create a pathway through which bullied individuals express their frustrations. Identifying and addressing anger rumination can serve as a pivotal step in mitigating the transition from victimization to perpetration of unethical behavior online.

### 4.4 The chain intermediation effect of relative deprivation and anger rumination in offline bullying and online unethical behavior

This study discovered that relative deprivation and anger rumination serve as dual mediators between offline bullying and cyber unethical behavior. Relative deprivation significantly fosters anger rumination, essentially suggesting that “a sense of inferiority leads to greater cynicism.” Anger rumination exacerbates individuals' feelings of anger, which, in turn, may lead to extreme and inappropriate actions online (Xue et al., 2011). Both relative deprivation and anger rumination amplify and sustain negative emotions following bullying and negative events. First, real-life victimization triggers anger rumination, as individuals fixate on the unfair treatment they have experienced. This rumination amplifies negative emotions and leads to a greater sense of perceived deprivation. Second, the heightened perception of deprivation motivates individuals to seek retribution or compensation, which may manifest as online unethical behavior.

Therefore, interventions for online unethical behavior should not only aim at providing immediate emotional relief to those offline bullied, but also require long-term support. This requires the collective attention and participation of families, schools and communities to promote a healthy online environment, establish appropriate norms and rules for online behavior, enhance the monitoring of online activities, reduce the likelihood of bullying among students, and to support them in responding appropriately to online misconduct. From the perspective of the individual college student, colleges and universities should provide anger management training and resources to help individuals regulate their emotions and reduce the tendency to ruminate about anger. Anger can be effectively managed through techniques such as psychocognitive behavioral therapy and positive thinking exercises (Deffenbacher et al., 2000).

## 5 Perspectives

The study examines the relationship between offline bullying behavior and online unethical behavior, examines the dual mediating roles of angry rumination and relative deprivation in this process, and validates the role of these two moral cognitive factors in this process. The study of the factors influencing individual online unethical behavior is a systematic and complex project, that is subject to the interaction of multiple factors. It is influenced by the individual's personality traits, moral cognition, emotions and feelings, and other internal factors, as well as external factors such as the offline social events in which the individual is involved, the online network events, the individual's family environment and peer relationships. Researchers can further study the following aspects: (1) systematically sort out and integrate intrinsic and extrinsic factors, examine their mechanisms and pathways of action on cyber immoral behavior, and elucidate the status and roles of different levels of factors in the causes of online unethical behavior. (2) Using big data technology to build a decision-making model of online moral behavior from multiple dimensions and levels, to improve the existing moral decision-making theory and enrich the theory of online moral decision-making. By collecting and analyzing the behavioral data of internet users on social media, forums, shopping websites and other platforms, it identifies the key factors that influence online moral decision-making and builds a computational model to predict online moral behavior. Big data analysis can offset the limitations of traditional questionnaire surveys and experimental studies and provide more comprehensive, objective and real-time behavioral insights. (3) Conduct tracking research on individual cybernetic behavior. The individual's online behavior is a dynamic developmental process, and his/her online behavior will continue to change with time, accumulation of experiences and maturity of knowledge. Future research can conduct longitudinal studies to continuously track the course of changes in individual online moral decision-making behavior, examine their developmental patterns, and provide a basis for developing targeted online moral education strategies. (4) Based on the results of the analysis of the influencing factors of network unethical behavior, we will explore diverse and effective intervention methods and means of promoting online ethical behavior, in order to develop a three-dimensional network ethics education strategy and improve the level of individual network ethics in all aspects.

## Data availability statement

The data that support the findings of this study are available in the figshare.com (https://doi.org/10.6084/m9.figshare.26061253).

## Ethics statement

The studies involving humans were approved by the Academic Ethics Committee of Zhongshan Institute belongs to the Zhongshan Institute of the University of Electronic Science and Technology. The studies were conducted in accordance with the local legislation and institutional requirements. The participants provided their written informed consent to participate in this study.

## Author contributions

YL: Writing – original draft. ZT: Writing – review & editing, Data curation. QX: Writing – review & editing.
